# Valorisation of industrial hemp (*Cannabis sativa* L.) residues and cheese whey into volatile fatty acids for single cell protein production

**DOI:** 10.1016/j.ese.2024.100439

**Published:** 2024-06-14

**Authors:** Carlo Moscariello, Silvio Matassa, Francesco Pirozzi, Giovanni Esposito, Stefano Papirio

**Affiliations:** Department of Civil, Architectural and Environmental Engineering, University of Napoli Federico II, via Claudio 21, 80125, Napoli, Italy

**Keywords:** Hemp biomass residues, Cheese whey, Volatile fatty acids, Acidogenic fermentation, Single cell protein

## Abstract

The production of single cell protein (SCP) using lignocellulosic materials stands out as a promising route in the circular bioeconomy transition. However, multiple steps are necessary for lignocellulosics-to-SCP processes, involving chemical pretreatments and specific aerobic cultures. Whereas there are no studies that investigated the SCP production from lignocellulosics by using only biological processes and microbial biomass able to work both anaerobically and aerobically. In this view, the valorisation of industrial hemp (*Cannabis sativa* L.) biomass residues (HBRs), specifically hurds and a mix of leaves and inflorescences, combined with cheese whey (CW) was investigated through a semi-continuous acidogenic co-fermentation process (co-AF). The aim of this study was to maximise HBRs conversion into VFAs to be further used as carbon-rich substrates for SCP production. Different process conditions were tested by either removing CW or increasing the amount of HBRs in terms of VS (i.e., two and four times) to evaluate the performance of the co-AF process. Increasing HBRs resulted in a proportional increase in VFA production up to 3115 mg HAc L^−1^, with experimental production nearly 40% higher than theoretical predictions. The synergy between HBRs and CW was demonstrated, proving the latter as essential to improve the biodegradability of the former. The produced VFAs were subsequently tested as substrates for SCP synthesis in batch aerobic tests. A biomass concentration of 2.43 g TSS L^−1^ was achieved with a C/N ratio of 5.0 and a pH of 9.0 after two days of aerobic fermentation, reaching a protein content of 42% (g protein per g TSS). These results demonstrate the overall feasibility of the VFA-mediated HBR-to-SCP valorisation process.

## Highlights

• Hemp residues (HBRs) and cheese whey (CW) were co-fermented in semi-continuous mode.
• An acetic acid concentration of up to 3115 mg L^−1^ was achieved with a HBRs:CW volatile solids ratio of 4:1.
• Acetic acid dominated the produced volatile fatty acids (VFAs), accounting for 66 and 93%.
• Up to 92% of the produced VFAs were aerobically converted into single cell protein (SCP).
• Up to 2.43 g L^−1^ total suspended solids of SCP, with a protein content of 42%, was achieved within two days.

## Graphical abstract

**Figure:**
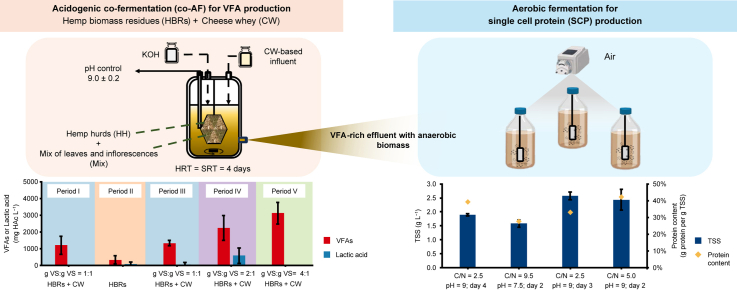

## 1 Introduction

The use of lignocellulosic biomass (e.g., agricultural residues) in second-generation biorefineries, in analogy to petroleum-based refineries, is being increasingly regarded as a promising approach for the sustainable production of biofuels/bioenergy and other value-added products such as biodegradable plastics, biochemicals, or high-quality protein-based products such as single cell protein (SCP) [1,2]. Considering the increase in the animal-based protein demand, currently reaching about 202 million tons year^−1^, and the fact that 1 kg of animal-based protein requires almost 6 kg of plant biomass, the current available resources will likely not be sufficient to guarantee sustainable livelihoods at the global level [3,4]. The production of SCP, and second-generation SCP using waste resources in particular, could overcome the environmental drawbacks of the conventional protein sources (e.g., meat, fish, chicken, or soybean) such as excessive use of land, fertilisers, energy and water, and greenhouse gases (GHGs) emissions, by using carbon and nitrogen from different residual organic streams [[3], [4], [5], [6], [7]]. For instance, the water footprint of particular SCP-based products (i.e., FeedKind protein, a bacterial SCP produced from natural gas or biogas) is reported to be about 20 and 140 times lower than that of fishmeal and soybean meal, respectively [8]. The use of SCP, expected to grow up to 22 million metric tons by 2035 [9], would also contribute to the achievement of the sustainable development goals (SDGs) promoted by the United Nations (UN). Specifically, SCP could respond to the goals of zero hunger and sustainable production and consumption, which aim to achieve food security and limit food and energy losses using innovative circular economy approaches [10,11].

SCP are produced typically through aerobic microbial fermentation processes using microorganisms such as yeasts, microalgae, fungi or bacteria [9,12]. Several inorganic or organic substrates can be used as precursors for SCP production, e.g., gaseous substrates rich in CH_4_, CO_2_, CO, or H_2_, or liquid substrates rich in organic compounds (e.g., sugars, organic acids). Several studies in literature reported SCP production from different lignocellulosic materials by fermentation of pretreated substrates or the derived sugar-rich hydrolysate, reaching a protein content up to 50% of the dry biomass [5]. In fact, despite the abundance and low cost of lignocellulosic biomass [13], its complex structure made of cellulose, hemicellulose, and lignin, with the latter that limits the bioconversion of such substrates [14], makes pretreatments (e.g., physical or chemical) necessary to enhance the conversion of lignocellulosic biomass by removing the lignin layer or by modifying the crystalline structure [15,16]. However, the use of pretreatments constitutes a problem from an economic point of view, i.e., capital and operational costs in real-scale applications [17], as well as due to the potential formation of toxic by-products during the pretreatment [18]. To overcome these limitations, the use of carbon-rich organic wastes as co-substrates to enhance the biodegradability of lignocellulosic biomass offers a valid alternative to the conventional pretreatments and fully aligns with the concept of circular bioeconomy [19,20]. In this view, further process symbioses can be realised by implementing the biological capture of carbon and upgrading nutrients recovered from biowastes obtained by combining anaerobic bioprocesses and aerobic fermentation to produce SCP [21]. The use of organic carbon-rich waste streams (e.g., underutilised or discarded by-products) in alternative to clean synthetic media containing sugar-based substrates can alleviate the issues related to the high cost of biomass production in large-scale production [22]. Findings in literature reported that volatile fatty acids (VFAs)-rich waste streams, derived from anaerobic digestion (AD) or acidogenic fermentation (AF) processes of highly biodegradable food waste, have the potential to be used as carbon source for SCP production in a sequential anaerobic-aerobic fermentation process [[22], [23], [24]]. Particularly, the AF process corresponds to the hydrolysis, acidogenesis, and acetogenesis stages of AD, where the organic substrates are converted into VFAs, alcohols, H_2_, and CO_2_.

Among the many available lignocellulosic substrates, this study focused on industrial hemp (*Cannabis sativa* L.) due to its high attractiveness for the emerging bio-based sector [17], and particularly on the hemp biomass residues (HBRs), i.e.*,* hemp hurds (HH), leaves and discarded inflorescences, which constitute the biggest portion of the hemp plant (≈60–80% w/w) [25]. Although often considered as wastes, HBRs could instead enable the synthesis of a wide spectrum of marketable products, spanning from biofuels (e.g., biodiesel, bioethanol, biomethane, or biohydrogen) to high-value biomaterials (e.g., biopolymers and SCP) [17,26]. The present study investigated the acidogenic co-fermentation (co-AF) of HBRs with another typically abundant organic waste such as cheese whey (CW), with the aim to maximise the production of VFAs to be further used as precursors for the production of SCP [17,27]. When lignocellulosic biomass is used in anaerobic fermentation processes, co-fermentation is generally used to balance the high carbon-to-nitrogen (C/N) ratio [28], macro and micro nutrients, pH, and dry matter content [29,30], enhancing both biohydrogen production and VFA accumulation. Concerning CW, this substrate represents the main waste of the dairy industry and is made of different dilutions of milk and washing water, resulting in an important organic load, i.e., up to 100 g L^−1^ of chemical oxygen demand (COD) [19,31]. Moreover, due to its high organic nitrogen (i.e., 0.2–5.4 g L^−1^), fats (i.e., 0.08–10.58 g L^−1^) and lactose (i.e., 0.18–60.00 g L^−1^) content [32,33], CW is considered a nutritional supplement and an optimal co-substrate to enhance the organic acids production from the fermentation of lactose [31] through its microorganisms, constituted mainly by lactic acid bacteria (LAB), and to further support the SCP production process [19]. According to Condon [34] and Zotta et al. [35], LAB can be classified as facultative anaerobic and O_2_-tolerant microorganisms that can easily switch from anaerobic to aerobic conditions and grow as long as they have a suitable substrate to be fed on.

To the best of the authors’ knowledge, the possibility of using LAB to produce VFAs under anaerobic conditions and further transform them into SCP under aerobic conditions has never been demonstrated so far. This could lead to several advantages, such as the possibility to valorise the anaerobically produced biomass or the recovery of VFAs without expensive techniques of extraction and purification (e.g., filtration membranes) or, in addition, the possibility to use the same mixed microbial cultures in both the anaerobic and aerobic processes. A previous study based on batch screening experiments indicated that the co-AF between HH or a mix of leaves and inflorescences (Mix), as source of HBRs, and CW as organic co-substrate, holds a promising VFA production potential, i.e., 379 ± 27 and 651 ± 65 mg of acetic acid equivalent per g of volatile solids added (mg HAc per g VS), respectively [26]. Nevertheless, the performances of the process under more technically relevant settings (e.g., presence of multiple HBRs, bioreactors operated in continuous/semi-continuous modes), as well as the identification of the optimal operating conditions to obtain SCP through the produced VFAs by using the same microbial biomass both under anaerobic and aerobic conditions requires further investigation. Given the above, after preliminary batch tests, the present study aimed to investigate the optimal conditions for VFA production within a semi-continuous anaerobic bioreactor using HBRs (i.e., HH and Mix) and CW as co-fermentation substrates. In this process, the continuous operating mode involved the influent (diluted CW) and the effluent, while the HBRs were fed in a batch mode. After an initial acclimation period, different operating conditions were tested to optimize VFA concentration and productivity. Particularly, the synergistic effect between HBRs and CW was evaluated by testing the HBRs individually or by increasing their amount (i.e., g VS) compared to CW. Finally, the liquid VFA-rich fermentate was used in batch tests to investigate the ability of the microbial biomass involved in the co-AF to grow under aerobic conditions and produce SCP. To this end, the effect of operational parameters such as pH, nitrogen source (i.e., organic and mineral N) and the C/N ratio on the VFAs-mediated HBRs-to-SCP conversion process was evaluated.

## 2 Materials and methods

### 2.1 Sources of hemp hurds, mix of leaves and inflorescences and cheese whey

The hemp biomass used in this work (i.e., HH and Mix) originated from a cultivation of *Cannabis sativa* L., cultivar “Eletta Campana”, located in the Campania region (Italy). The cultivation, harvesting, and storage conditions were the same as Moscariello et al. [26] reported. The HH and Mix substrates, harvested and collected in two different periods, are hereafter identified as HH_A_ and HH_B_ and Mix_A_ and Mix_B_, respectively. The characteristics of HH and Mix, which slightly changed between the two periods, are reported in Table 1. As a co-substrate, fresh CW from a cow cheese factory located near Naples (Italy) was collected in three different periods (i.e., CW_A_, CW_B_, and CW_C_) and used for the batch and the semi-continuous co-AF (Table 1). The CW was immediately stored at −20 °C before being used in the experimental activity. HBRs were characterised in terms of total (TS) and volatile solids (VS), while CW in terms of TS, VS, soluble COD (sCOD), ammonium nitrogen concentration (N–NH_4_^+^), pH, VFA, and lactic acid (LA) concentrations (Table 1).

**Table 1:** Characterisation of the anaerobic inoculum, cheese whey (CW), hemp hurds (HH) and mix of leaves and inflorescences (Mix). TS: total solids; VS: volatile solids; sCOD: soluble chemical oxygen demand; N–NH_4_^+^: ammonium nitrogen concentration; VFAs: volatile fatty acids; LA: lactic acid. All values are expressed as the mean and standard deviation of three replicates.

Substrate	Type	Pretreatment	TS (%)	VS (%)	pH	sCOD (g L^−1^)	N–NH_4_^+^ (g L^−1^)	VFAs (mg HAc L^−1^)	LA (mg HAc L^−1^)
Inoculum	n.a.^a^	None	6.91 ± 0.18	4.93 ± 0.18	7.80	56.53 ± 1.33	3.78 ± 0.03	n.a.	n.a.
	n.a.^a^	Thermal^b^	5.71 ± 0.05	3.38 ± 0.07	9.90	18.15 ± 0.35	2.07 ± 0.03	80.60 ± 3.98	65.11 ± 7.68
	n.a.^a^	Thermal + manual filtration	5.95 ± 0.24	3.71 ± 0.01	10.00	7.74 ± 0.09	1.64 ± 0.04	80.60 ± 3.98	65.11 ± 7.68
CW	A	None	3.53 ± 0.01	3.16 ± 0.01	4.60	53.64 ± 0.60	0.37 ± 0.02	3598 ± 176	17144 ± 474
	B	None	2.79 ± 0.09	2.41 ± 0.09	4.55	29.30 ± 0.30	0.04 ± 0.00	1187 ± 294	2729 ± 108
	C	None	5.02 ± 0.25	4.59 ± 0.24	4.59	40.40 ± 0.57	0.04 ± 0.03	608 ± 18	2101 ± 19
HH	A	None	94.20 ± 4.20	86.62 ± 3.24	n.a.	n.a.	n.a.	n.a.	n.a.
	B	None	88.20 ± 0.09	73.00 ± 5.50	n.a.	n.a.	n.a.	n.a.	n.a.
Mix	A	None	87.81 ± 0.18	69.58 ± 0.19	n.a.	n.a.	n.a.	n.a.	n.a.
	B	None	85.40 ± 1.30	66.30 ± 2.30	n.a.	n.a.	n.a.	n.a.	n.a.

a n.a.: not available.

b Thermal: 105 °C for 4 h.

### 2.2 Source and pretreatment of anaerobic inoculum

A bovine digestate originating from a full-scale anaerobic digester near Salerno (Italy) was used as source of anaerobic inoculum for the co-AF tests. A thermal shock pretreatment was used to improve hydrolysis, achieve pasteurisation by killing most of pathogenic bacteria, and inhibit the methanogenic archaea, thus ensuring the required AF conditions [36]. The inoculum was pretreated at 105 °C for 4 h [37], since longer times may result in the loss of easily fermentable sugars [38]. Prior to the thermal pretreatment, the inoculum was manually filtered through a burlap bag with a fine mesh (diameter of about 1 mm) to eliminate the bigger particles that could have hindered the proper functioning of the pumping system. The thermally pretreated inoculum was characterised similarly to the CW (see section 2.1) prior to being used in the co-AF tests (Table 1).

### 2.3 Batch acidogenic co-fermentation tests

Preliminary batch co-AF tests were used to investigate the operating conditions that led to the maximum VFA production in the shortest time and to identify the best parameters to be used in the semi-continuous process (see section 2.4). Both HH_A_ and Mix_A_ were used as HBR sources (Table 1). Particularly, four co-AF tests were performed: (1) Mix + CW with an initial pH of 9.90 (T_1_); (2) HH + CW with an initial pH of 9.90 (T_2_); (3) Mix + CW with an initial pH of 8.50 (T_3_); (4) HH + CW with an initial pH of 8.50 (T_4_). HH_A_ and Mix_A_ were the HBRs used, while CW_A_ was the substrate used for CW. T_1_ and T_2_ lasted 21 days, while T_3_ and T_4_ lasted 14 days.

The co-AF tests were performed in a 2.2 L (working volume) borosilicate glass reactor with a design similar to that adopted by Oliva et al. [39]. Mesophilic conditions (i.e., T = 37 ± 2 °C) were guaranteed through an ED (v.2) heating bath (Julabo, Germany) connected to the reactor water jacket. The reactor was constantly stirred through a magnetic stirrer. A stainless steel mesh container (15 × 10 × 10 cm) was used to contain the HBRs [39], with a grid able to hold them and avoid an excessive dispersion of the smallest pieces into the liquid phase, thus also simplifying the sampling phase and allowing the removal of the HBRs from the reactor during the semi-continuous operation (see section 2.4). The anaerobic inoculum was poured into the reactor for 60% of the working volume (i.e., 1.32 L), and HBRs and CW were added by maintaining an inoculum-to-substrate (I/S) ratio of two in terms of VS (i.e., 2 g VS Inoculum per g VS (HBRs + CW)). Specifically, 22.33 g VS were used for HBRs + CW, dosing 50% of the added VS from the HBRs and 50% from the CW. Thus, the amounts of HBRs were 16.05 and 12.89 g for Mix and HH, respectively, while 354 mL of CW was added for each test. The pH of the CW was adjusted to neutrality (≈7.00) with a 3 M NaOH solution prior to being used in the tests. Finally, tap water was added to reach the final working volume. Concerning T_3_ and T_4_, the initial pH of the inoculum was adjusted to 8.50 with hydrochloric acid (HCl 37% v/v). After filling the reactor, its headspace was flushed with Argon gas to ensure the anaerobic conditions. The liquid fermentate was sampled eight times for T_1_ and T_2_ and five times for T_3_ and T_4_. The net VFA and LA productions for each test were calculated by subtracting those of the anaerobic inoculum alone based on the data reported by Moscariello et al. [26].

### 2.4 Semi-continuous acidogenic co-fermentation

The investigation of the semi-continuous co-AF process started after the batch tests and lasted 137 days, aiming at the concomitant valorisation of both HH and Mix with CW into VFAs. The reactor configuration, stirring and heating conditions were similar to those described in section 2.3. Particularly, HH and Mix were dosed considering the average HBRs composition (i.e., 61% and 39% (w/w), respectively) [40], and fed in batch mode through the steel cage setting a solid retention time (SRT) of four days. The continuous-flow reactor operations, instead, involved the liquid influent and effluent and were characterized by a hydraulic retention time (HRT) of four days (i.e., the same as the SRT), based on the data obtained in the screening batch co-AF tests (see section 2.3). For this aim, the liquid phase was continuously fed to and removed from the reactor through a Reglo ICC Indipendent Channel peristaltic pump (EN.CO., Italy) with a flow rate of 0.38 mL min^−1^.

Five different experimental periods were defined (Table 2). During periods I and III, the co-AF of the HBRs and CW with the same VS concentration (i.e., 12.24 g VS L^−1^, Table 2) was investigated, while the CW feeding was interrupted during period II, and the liquid influent was replaced by tap water amended with a micronutrient solution (0.01% v/v), prepared according to Sun et al. [41], and N–NH_4_^+^ at the same concentration detected in the CW (Table 1). This was done to evaluate the effect of the lack of CW on VFA production, avoiding the continuous addition of carbon and microorganisms through the influent. For periods IV and V, instead, the mass of VS provided through the HBRs was two and four times higher, respectively, than that of the CW, which was maintained.

**Table 2:** Experimental conditions tested during the semi-continuous co-AF. g VS: gram of volatile solids of HBRs and CW added; OLR: organic loading rate of HBRs and CW; g VS L^−1^: gram of volatile solids added per litre of influent.

Periods	Days	HBRs			CW	
		Type	(g VS)	OLR (g VS L^−1^ d^−1^)	Type	OLR (g VS L^−1^ d^−1^)
I	1–24	HH_A_ + Mix_A_	12.24	0.79	CW_A_ - CW_B_	3.06
II	25–50	HH_A_ + Mix_A_	12.24	0.79	n.a.^a^	n.a.^a^
III	51–66	HH_A_ + Mix_A_	12.24	0.79	CW_B_	3.06
IV	67–107	HH_A_ + Mix_A_, Mix_B_	24.48	1.85	CW_B_	3.06
V	108–137	HH_B_ + Mix_B_	48.96	6.35	CW_B_ - CW_c_	3.06

a n.a.: not available.

The initial pH was set equal to 8.50 ± 0.20 by means of HCl (37% v/v) addition and, from day 22, changed and maintained at 9.00 ± 0.20 with an alkaline solution of potassium hydroxide (KOH 1.5 M) to contrast methanogenesis. A pH electrode (VWR, USA) was used to monitor the pH evolution, while a pH/ORP 300 controller (Cole-Parmer, USA) coupled to an EVO45 pump (Verderflex, UK) [39] dosing the KOH solution was used to control the pH. Prior to start and after each HRT, when the reactor was opened and the spent HBRs were replaced with the fresh substrate, the headspace of the reactor was flushed with Argon to ensure the anaerobic conditions. The effluent was sampled every two to four days and analysed in terms of total suspended solids (TSS), sCOD, N–NH_4_^+^, VFA, and LA concentrations.

### 2.5 Batch aerobic fermentation tests

Batch aerobic fermentation tests were used to investigate the ability of the microbial biomass involved in the semi-continuous co-AF of HBRs and CW to grow under aerobic conditions and synthesize protein-rich biomass, i.e., SCP. These tests were performed with different C/N ratios and pH values, as well as by using CW with or without the addition of ammonium nitrogen, thereby evaluating the use of the sole organic N, or its combination with mineral N, as nitrogen sources. The batch tests were conducted by using the VFA-rich liquid fermentate of the co-AF process sampled in the different experimental periods (Table 3) as the carbon source for SCP synthesis. B_1_ and B_2_ were conducted by modifying the initial pH of the fermentate, while the C/N ratio, calculated as the ratio between the total organic carbon (TOC) and the total Kjeldahl nitrogen (TKN), corresponded to the values originally present in the fermentate. B_3_ and B_4_, instead, were conducted by modifying the C/N ratio, calculated as the ratio between the TOC and the N–NH_4_^+^ supplemented in the form of ammonium chloride (NH_4_Cl) and without altering the initial pH value. In all tests, phosphorus (P) as potassium phosphate (K_2_HPO_4_), added according to a N/P ratio of 5.0 [42], and a micronutrients solution (0.01% v/v) (see section 2.4), were provided to ensure a balanced nutrient supply. An antifoam agent (Antifoam 201, Sigma Aldrich) was used to avoid the possible formation of foam during the aeration [5]. The VFA-rich liquid fermentate pH adjustment was performed using HCl (37% v/v) addition.

**Table 3:** Characterisation of the VFA-rich liquid fermentate used for the aerobic batch test. TOC: total organic carbon; TKN: total Kjeldahl nitrogen; N–NH_4_^+^: ammonium nitrogen concentration; VFAs: volatile fatty acids; LA: lactic acid; sCOD: soluble chemical oxygen demand; TSS: total suspended solids.

Test	C/N		pH		Co-AF experimental period	TOC (mg L^−1^)	TKN (mg L^−1^)	N–NH_4_^+^ (mg L^−1^)	VFAs (mg HAc L^−1^)	LA (mg HAc L^−1^)	sCOD (mg L^−1^)	TSS (g L^−1^)
	Original^a^	Modified^b^	Original	Modified^c^								
B_1_	2.5	n.a.^d^	9.0	n.a.	Period IV	1415	569	68.49	2896	464	3216	0.28
B_2_	9.5	n.a	9.0	7.5	Period IV	1502	159	43.94	1607	390	4234	0.36
B_3_	9.5	2.5	9.0	n.a.	Period IV	2547	268	94.67	2900	1550	6026	0.50
B_4_	9.5	5.0	9.0	n.a.	Period V	3838	404	108.70	4829	829	9625	0.18

a Calculated as TOC/TKN.

b Calculated after the NH_4_Cl addition as TOC/N–NH_4_^+^.

c Modified with HCl (37 % v/v) addition.

d n.a.: not available.

The tests were performed in triplicate in 250 mL Schott bottles, filled with 150 mL of the VFA-rich liquid fermentate, and kept under mesophilic conditions (i.e., T = 37 ± 2 °C). The aeration was guaranteed by air pumps (Aquarium systems, NEWA, Italy), and the air was continuously sparged in the fermentate through aeration stones with a nominal flowrate of 190 L h^−1^. After each sampling, fresh liquid fermentate was added in the same amount to maintain a constant volume. The consumption of VFAs, the biomass growth in terms of TSS concentration, the sCOD and the N–NH_4_^+^ consumption, and the protein content (i.e., g protein per g TSS) were the parameters used to monitor the process.

### 2.6 Analytical procedures

The TS and VS of the inoculum, the HBRs, and the CW, as well as the TSS of the effluent, were analysed according to the Standard Methods [43]. According to a recent study [26], under similar co-AF conditions the biogas production constitutes only a small part of the chemical energy in the form of COD if compared to VFAs (≈0.7–4.8% mg H_2_-COD per mg VFAs-COD). Thus, the volume of biogas produced was not monitored, and only its composition was analysed daily through a Star 3400 gas chromatograph (Varian, USA) to control the possible biomethane formation. VFA and LA analyses were performed using high-performance liquid chromatography (HPLC) using a UVD 340U HPLC system (Dionex, USA) equipped with a diode array detector and a Metrosep organic acid 250/7.8 column (Metrohm, Switzerland), according to the procedure described by Moscariello et al. [26]. sCOD was analysed through the closed reflux colourimetric method [43], the N–NH_4_^+^ concentration was measured with the blue indophenol method [44], and the TOC was determined through a TOC-L analyser (Shimadzu, Japan). The protein content of the produced SCP was measured on the biomass harvested through centrifugation at 6000 rpm for 10 min and washed in a 0.9% NaCl saline solution. The organic nitrogen content of the biomass was measured as TKN [43], and a conversion factor of 6.25 was applied to calculate the final protein concentration [33,45].

### 2.7 Calculations

The total VFA production was calculated as the sum of the concentrations of the single organic acids detected at each sampling time. Short-chain fatty acids such as formic, acetic, propionic, butyric, isovaleric, valeric and caproic were considered in this study.

The VFA productivity (Π) represents the amount of VFA produced in the unit of time per unit of working volume (mg HAc L^−1^ d^−1^). Π has been calculated based on equation (1):

(1) $$\Pi_{i}=\frac{\left[V_{\text{out}}\times \frac{\left({VFA}_{i}+{VFA}_{i-1}\right)}{2}+V_{\text{TOT}}\times \left({VFA}_{i}-{VFA}_{i-1}\right)\right]}{V_{\text{TOT}}}\times \frac{1}{t_{i}}$$

where V_OUT_ represents the volume of the effluent in the reference time, and V_TOT_ represents the working volume. The subscripts *i* and *i*−1 indicate the values of the VFA production at the chosen sample time (t) and at the previous one, respectively.

To calculate the potential synergistic effect of co-fermenting HBRs and CW, the theoretical VFA production obtained only through CW (VFA(CW)) was estimated based on equation (2):

(2) $$\text{VFA}\left(\text{CW}\right)=\frac{\left[\text{VFA}\left(\text{Period}\;I\right)-\text{VFA}\left(\text{HBRs}\right)\right]+\left[\text{VFA}\left(\text{Period}\;\text{III}\right)-\text{VFA}\left(\text{HBRs}\right)\right]}{2}$$

where VFA (Period I) and VFA (Period III) refer to the mean net VFA productions along the two experimental periods employing CW and HBRs at a VS:VS ratio of 1:1, while VFA(HBRs) refers to the mean VFA production measured on period II when the supply of CW was interrupted.

The above-mentioned parameters were then used to calculate the theoretical VFA production for periods IV and V based on equations (3), (4):

(3) VFA(PeriodIV)=VFA(CW)+2VFA(HBRs)

(4) VFA(PeriodV)=VFA(CW)+4VFA(HBRs)

These theoretical values were compared to the net VFA production measured in periods IV and V.

Finally, for the batch aerobic tests, the biomass yield coefficient (Y), i.e., the amount of biomass produced per unit of substrate consumed (i.e., sCOD) [33], was calculated according to equation (5):

(5) $$Y=\frac{Net\;biomass\;growth}{{sCOD}_{\text{IN}}-\;{sCOD}_{\text{OUT},i}}$$

where ${sCOD}_{\text{IN}}$ refers to the sCOD value at the beginning of each test (i.e., day 0), and ${sCOD}_{\text{OUT},i}$ refers to the value of the sCOD at the chosen sampling time *i*.

## 3 Results and discussion

### 3.1 Volatile fatty acids production in batch co-AF tests

**Fig. 1:**
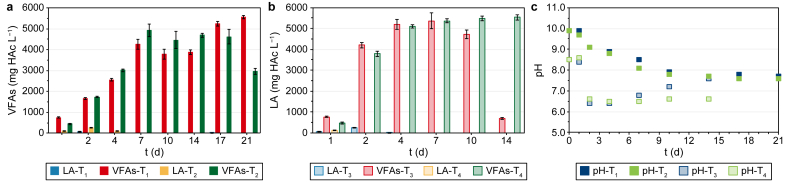
Evolution of the net VFA and LA (**a**–**b**) production and pH (**c**) during the four batch co-AF tests. The net VFA and LA (**a**–**b**) productions, expressed as acetic acid equivalent (HAc) per litre, were obtained by subtracting the VFA and LA productions of the sole inoculum [26]. Each value is the mean of three replicate measurements for each sampling time, and bars indicate the standard deviation. T_1_: Mix + CW with pH = 9.90; T_2_: HH + CW with pH = 9.90; T_3_: Mix + CW with pH = 8.50; T_4_: HH + CW with pH = 8.50.

In Fig. 1, the net VFA and LA production (Fig. 1a and b), as well as the pH trend (Fig. 1c) during the four batch co-AF tests (i.e., T_1_, T_2_, T_3_, T_4_) are shown.

Concerning T_1_ and T_2_ (Fig. 1a), the VFA production continuously increased from day one to day seven, reaching a maximum value of 4251 ± 243 and 4922 ± 298 mg HAc L^−1^, respectively, on day seven, and then settled and decreased until day 21, with the only exception of Mix + CW (T_1_) on day 17. This trend is reflected by the decrease of pH during the first seven days (Fig. 1c) from an initial value of 9.90 for both T_1_ and T_2_ to 8.50 and 8.10 on day 7 (T_1_ and T_2_, respectively). Subsequently, the pH remained stable at a mean value of 7.70, reaching values where methanogenesis could be activated again [46]. The high starting pH values could probably be attributed to the effect of the thermal pretreatment. The T increase during the thermal shock pretreatment may have affected the dissociation of the NH_3_ in the digestate. Particularly, ammonium bicarbonate (2NH_4_HCO_3_) is easy to decompose by heating, thus forming ammonium carbonates ((NH_4_)_2_CO_3_) and removing CO_2_, with a result of a spontaneous increase of the pH value (Table 1) [47].

A fermentation process typically has a shorter duration than anaerobic digestion, depending on the type of substrate used. When only simple carbohydrates (e.g., glucose) need to be hydrolysed, AF lasts a few hours (4–12 h) [48], while for lignocellulosic materials, which have a lower biodegradability, the process duration can be extended up to 10–14 days, beyond which methanogenesis usually occurs, resulting in VFA consumption [49]. In this study, a decrease in VFA concentration probably linked to the production of CH_4_ occurred over the last four days (i.e., between day 17 and day 21), as proven by the biogas composition (i.e., 78–82% of CH_4_ for T_1_ and T_2_, respectively) (data not shown). In view of the possible full-scale development of the process, it was decided to consider the optimum VFA production achievable within seven days, since then it remained rather stable. These first two tests also proved the suitability of HH to produce VFAs in co-AF with CW, differently from what Moscariello et al. [26] reported in a previous study. In fact, regarding VFA production, on day seven HH (T_2_) showed comparable performances with Mix (T_1_), probably due to a better adaptation of the microbial cultures under these operating conditions.

T_3_ and T_4_ were meant to evaluate the effect of a lower initial pH of 8.50, corresponding to the value observed on day seven of the previous T_1_ and T_2_. For both T_3_ and T_4_, the VFA production rapidly increased in the first two days, reaching values of 5212 ± 252 and 5106 ± 75 mg HAc L^−1^ for Mix and HH, respectively, on day four (i.e., +51 (T_3_) and +41% (T_4_) than T_1_ and T_2_, respectively, on the same day) (Fig. 1b). The VFA production on day seven was only 3% and 5% higher for T_3_ and T_4_, respectively, than that obtained on day four and remained constant for HH or decreased for Mix in the subsequent period. Concerning pH (Fig. 1c), it rapidly decreased to 6.50 in the first two days because of the high amount of VFAs produced for both T_3_ and T_4_, while it remained constant for T_4_ and increased up to 7.50 for T_3_ by the end of the tests. For T_3_, four days seemed sufficient to obtain the highest production of VFAs, after which VFAs were probably converted to CH_4_ and CO_2_ with decreased measured concentrations and increased pH to values more favourable for methanogenesis. Thus, as seen in T_1_ and T_2_, also for T_3_ and T_4_ the co-AF process showed comparable performances for Mix and HH, with the latter that, although being less biodegradable than Mix due to a lower surface area and bigger dimensions [40], showed a similar VFA production rate up to four days.

**Fig. 2:**
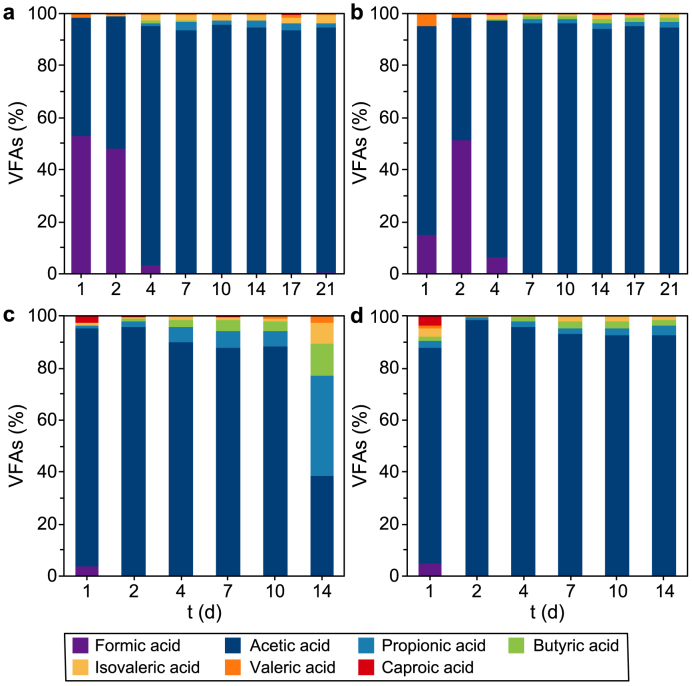
Speciation of the VFAs produced during the four co-AF batch screening tests. The percentage of the acids for each experimental condition has been calculated as the ratio between the single acid and the total VFAs measured. All values refer to the mean of three replicate measurements for each sampling day. **a**, T_1_: Mix + CW with pH = 9.90; **b**, T_2_: HH + CW with pH = 9.90; **c**, T_3_: Mix + CW with pH = 8.50; **d**, T_4_: HH + CW with pH = 8.50.

The VFA speciation (Fig. 2) shows how the most abundant VFA was acetic acid under each experimental condition, confirming what reported in literature for alkaline pH fermentation [50] and what was previously observed by Moscariello et al. [26]. Particularly, the highest acetic acid content for T_1_ and T_2_ (i.e., 94% and 96% on the total VFAs, respectively) was registered on day seven, while for T_3_ and T_4_ (i.e., 90% and 96% on the total VFAs, respectively) on day four.

### 3.2 Volatile fatty acids production in semi-continuous co-AF

The results of the batch tests (section 3.1) were used to set the HRT and the SRT for the semi-continuous reactor. The HRT of the influent (i.e., CW diluted in tap water) and the SRT of the HBRs were set equal to four days.

**Fig. 3:**
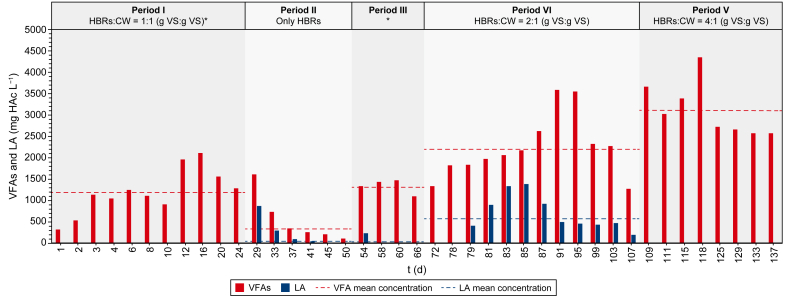
Evolution of the net VFA and LA productions obtained during the 137 days of semi-continuous co-AF of HBRs (i.e., HH and Mix) and CW. VFA and LA productions are expressed as concentrations of acetic acid equivalent (mg HAc L^−1^) and were obtained by subtracting the VFAs and the LA of the influent for each day from the effluent. The mean of the net VFA and LA productions for the corresponding period is reported. Period I: co-AF of HBRs and CW_A_ with HBRs:CW = 1:1 (g VS per g VS); Period II: AF of HBRs without CW_B_; Period III: co-AF of HBRs and CW_B_ with HBRs:CW = 1:1 (g VS per g VS); Period IV: co-AF of HBRs and CW_B_ with HBRs:CW = 2:1 (g VS per g VS); Period V: co-AF of HBRs and CW_B_ and CW_C_ with HBRs:CW = 4:1 (g VS per g VS).

**Fig. 4:**
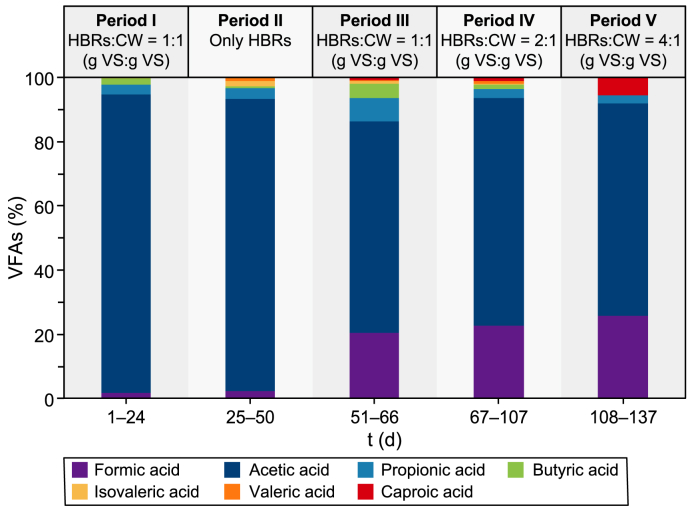
Speciation of VFAs during the five periods of semi-continuous co-AF of HBRs and CW. The percentage of each acid has been calculated as the ratio between the single acid and the total VFAs measured. The reported values are expressed as the mean of each experimental period. Period I: co-AF of HBRs and CW_A_ with HBRs:CW = 1:1 (g VS per g VS); Period II: AF of HBRs without CW_B_; Period III: co-AF of HBRs and CW_B_ with HBRs:CW = 1:1 (g VS per g VS); Period IV: co-AF of HBRs and CW_B_ with HBRs:CW = 2:1 (g VS per g VS); Period V: co-AF of HBRs and CW_B_ and CW_C_ with HBRs:CW = 4:1 (g VS per g VS).

In Fig. 3, the daily net and the mean VFA and LA productions during the different experimental periods are shown. The speciation of the VFAs produced during each experimental period is shown, instead, in Fig. 4.

#### 3.2.1 Periods I–II: effect of CW on VFA production

Fig. 3 shows how the semi-continuous co-AF process was subjected to two different trends in the VFA production. Particularly, a decreasing VFA production occurred between periods I and II, followed by a continuous increase from period III to period V. The pH of the process, which was initially at a value of 8.50 ± 0.20, was increased and maintained at 9.00 ± 0.20 from day 22 onwards after that CH_4_ was detected (i.e., up to 84%) in the produced biogas (data not shown). After an initial acclimation period for the microorganisms present in the anaerobic inoculum and in the influent CW, the VFA production on period I reached a mean value of 1202 mg HAc L^−1^ (Fig. 3) with a volumetric productivity of 457 mg HAc L^−1^ d^−1^, and a mean biomass concentration of 1.15 g TSS L^−1^ (Fig. S1, Supplementary Materials). The mean acetic acid content achieved approximately 93% of the produced VFAs (Fig. 4). Concerning the sCOD (Fig. S2, Supplementary Materials), which can be seen as an indicator of the extent of hydrolysis of the solid organic substrates [51], it reached a mean concentration of 3930 mg L^−1^ (Table S1, Supplementary Materials). Despite an increasing trend in the VFA production, the decreasing trends of the sCOD and of the biomass concentration in period I (Figs. S2 and S1, respectively, Supplementary Materials) were probably due to the partial washout under continuous liquid influent feeding and effluent withdrawal of the anaerobic inoculum initially fed to the reactor.

Concerning LA, despite the high amount present in the influent (i.e., 3024 ± 84 mg HAc L^−1^), no LA was detected in the effluent, meaning that it was probably converted into VFAs, particularly into acetic, propionic and butyric acids through the lactate-consuming pathway [52,53], or used for biomass synthesis. When lactose is the main substrate (e.g., in the influent CW), it can be hydrolysed into hexose sugars (i.e., glucose and galactose) and then converted into lactate or LA through homolactic or heterolactic transformations. The homolactic pathway leads to the formation of LA as a single product, while the heterolactic further converts LA into VFAs and CO_2_ [52,54]. LA was probably no longer detected in the system due to the onset and prevalence of metabolic pathways different from the homolactic one (e.g., heterolactic and/or other acidogenic fermentation pathways), leading to LA consumption.

After the first experimental period, it was decided to investigate the acidogenic fermentation potential of the system only in the presence of HBRs and without the source of carbon and new microorganisms continuously entering through CW. Thus, the CW was removed from the system in period II. The mean VFA and LA productions for this period were 332 and 89 mg HAc L^−1^ (Fig. 3), respectively, with the former being 72% lower than that of period I (i.e., 1202 mg HAc L^−1^). Also, the VFA productivity (i.e., 136 mg HAc L^−1^ d^−1^) was 70% lower than in period I (i.e., 457 mg HAc L^−1^ d^−1^). The lack of CW was also reflected in a mean sCOD concentration (i.e., 1050 mg L^−1^), which was 73% lower than that of period I (i.e., 3930 mg L^−1^) (Fig. S2, Supplementary Materials). Most probably, under these operating conditions, a partial washout of the microbial community carrying out the co-AF process, combined with a lower amount or readily bioavailable carbon and new microorganisms entering the system through CW, led to a decrease in all main process performance indicators, particularly in terms of VFA production until 112 mg HAc L^−1^ on day 50 (Fig. 3) (i.e., −93% than on day 24), possibly indicating that the presence of CW is essential to increase the biodegradability and the enzymatic hydrolysis of the HBRs [26,55]. The latter assumption finds further evidences in the increase of the average VS/TS ratio of the spent HBRs (i.e., those collected every four days) (Table S1, Supplementary Materials) observed between periods I and II (i.e., 86.5% and 88.5%, respectively), showing a lower hydrolysis capacity of the HBRs in period II if compared to period I with CW. Contrary to period I, LA was detected with a mean concentration of 89 mg HAc L^−1^ in period II, probably due to the microbial conversion of some lignocellulosic sugars such as hexose or pentose [56,57]. In the other periods, the presence of CW and its microorganisms (i.e., LAB), may have probably led to the conversion of LA into VFAs, avoiding its accumulation into the system. In period II, the lack of microorganisms continuously entering the system through CW probably stopped the conversion of LA into VFAs, leading to its accumulation, differently from what was seen in the previous period. Period II was terminated when the biomass concentration decreased to 0.25 g TSS L^−1^ (Fig. S1, Supplementary Materials), which was 78% lower than in period I.

#### 3.2.2 Period III: reintroduction of CW

The reintroduction of the CW in the influent in period III improved the mean VFA production (i.e., 1332 mg HAc L^−1^), which was 11% higher than that obtained in period I (i.e., 1202 mg HAc L^−1^) and four times higher than that of period II (i.e., 332 mg HAc L^−1^) (Fig. 3). The sCOD concentration also grew along this period (Fig. S2, Supplementary Materials) with a mean concentration of 2568 mg L^−1^, 40% higher than in period II and 35% lower than in period I due to a lower influent sCOD (Table S1, Supplementary Materials). Concerning the VFA speciation (Fig. 4), although acetic acid was confirmed as the prevailing among the total VFAs (i.e., ≈66%), formic acid started to be detected, reaching about 20% of the total VFAs, probably implying a possible evolution of the microbial community [58]. The biomass concentration (Fig. S1, Supplementary Materials) increased by 24% compared to period II (i.e., 0.31 and 0.25 g TSS L^−1^ for period III and II, respectively). Subsequently, it seemed to stabilise, probably meaning that the process started to be driven only by the microorganisms present in the CW from period III onwards. Despite an almost similar VFA production (Fig. 3), by comparing period III with period I, a 73% lower biomass concentration (i.e., 0.31 and 1.15 g TSS L^−1^ for period III and period I, respectively) (Fig. S1, Supplementary Materials) and a 27% lower VFA productivity than in period I (i.e., 333 and 457 mg HAc L^−1^ d^−1^ for period III and period I, respectively) can be observed. The presence of the anaerobic inoculum in period I, in fact, is the most reasonable explanation for the higher biomass concentration (see section 3.2.1) as well as the higher VFA productivity due to a possible synergy between the LAB present in the CW and the anaerobic bacteria present in the anaerobic inoculum [59], which probably missed from period II onwards due to an almost complete washout. Finally, the VS/TS ratio (Table S1, Supplementary Materials) was 4% lower (i.e., 84.8%) than in period II (i.e., 88.5%), probably implying a better hydrolysis of the HBRs due to the reintroduction of the CW in the system.

#### 3.2.3 Periods IV–V: increase of the HBR amount

The investigation about the possible synergistic effect in the VFA production between CW and HBRs was repeated in the last two periods (i.e., IV and V), by increasing the amount of VS from HBRs, respectively two and four times in period IV and period V (Table 2), while maintaining constant that of CW. The mean VFA production increased with the increase of the HBRs amount, reaching a mean production of 2235 mg HAc L^−1^ in period IV (i.e., 68% higher than the production of period III) (Fig. 3). After a problem with the pH control (i.e., pH decreased until 4.30) on day 79, the mean LA production increased (i.e., 584 mg HAc L^−1^), being almost ten times higher than in period III (i.e., 59 mg HAc L^−1^) (Fig. 3). Particularly, after the drop of pH, the process likely turned towards metabolic pathways different from the homolactic one, leading to LA accumulation rather than its conversion into VFAs. Proportionally, the increase in the VFA production corresponded to an increase also in the VFA productivity (i.e., 624 mg HAc L^−1^ d^−1^), which was 87% higher than that of period III (i.e., 333 mg HAc L^−1^ d^−1^).

The further increase of the HBRs in period V led to an increase in both VFA production (i.e., 3115 mg HAc L^−1^) and productivity (i.e., 819 mg HAc L^−1^ d^−1^), which were 39% and 31% higher than in period IV, respectively. Moreover, no LA was detected in this experimental period, meaning it was probably converted into VFAs. This trend occurred with almost the same concentration of biomass (i.e., 0.25 and 0.31 g TSS L^−1^ for periods V and IV, respectively) (Fig. S1, Supplementary Materials). Concerning the sCOD (Fig. S2, Supplementary Materials), the transition between period III and period V showed that, as the amount of HBRs increased, the sCOD also increased up to 8046 mg L^−1^ for period V (Table S1, Supplementary Materials), implying a potential improvement in the hydrolysis capacity of the system, as also shown by the decrease of the VS/TS ratio being 2% lower than in period III (Table S1, Supplementary Materials).

Overall, by considering that the mean VFA production of the HBRs in the absence of CW (period II) was 332 mg HAc L^−1^ (Fig. 3) and by further assuming it constant also during the VFA production in periods I and III with CW (i.e., 1202 and 1332 mg HAc L^−1^, respectively), it can be calculated that CW could have contributed with a mean VFA production of 935 mg HAc L^−1^ (see section 2.6 and Table S1, Supplementary Materials). Thus, with the increase in the amount of VS from HBRs in period IV and V (i.e., two and four times the VS of CW), the experimental production observed in the same periods (i.e., 2235 and 3115 mg HAc L^−1^ for period IV and V, respectively) was 40% and 38% higher than the productions theoretically obtainable (i.e., 1599 and 2264 mg HAc L^−1^ for period IV and V, respectively) (see section 2.6 and Table S1, Supplementary Materials). Therefore, as also reported by Moscariello et al. [26], the difference between the theoretical and the experimental VFA production observed in periods IV and V could prove the synergistic effect between the HBRs and CW, and could indicate the condition of period V (i.e., 4:1 g VS (HBRs):g VS (CW)) as the best performing one.

The comparison of the VFA production obtained in period V (i.e., 3115 mg HAc L^−1^) with those of many lignocellulosic substrates in similar processes proves the effectiveness of the process under the tested experimental conditions. Concerning the HBRs, the VFA production obtained in period V was 34% and 379% higher than the one obtained from a continuous rumen-mimetic process with rice straw (i.e., 2087 mg HAc L^−1^) and Japanese cedar (i.e., 655 mg HAc L^−1^) [60], respectively, and 47% higher than the VFA production obtained from an AF of corn stalk after an acid pretreatment (i.e., 2135 mg HAc L^−1^) [61]. Kim et al. [62] and Islam et al. [63] obtained almost the same VFA production from reed and sorghum stalks (i.e., 3112 and 3581 mg HAc L^−1^, respectively) after lime and an acid pretreatment, respectively, yet working both in batch conditions and thus implying VFA concentrations generally higher than those obtainable in a continuous system as the one adopted in this study. On the other hand, the VFA production of period V (i.e., 3115 mg HAc L^−1^) was 227% and 44% lower than the ones obtained by Song et al. [64] and Wang et al. [65], respectively, from grass clipping (i.e., 10226 mg HAc L^−1^) and corn stover (i.e., 4525 mg HAc L^−1^), with the latter that refers to a batch experiment. It is worth noting that almost all the VFA yields reported were obtained after chemically pretreating the lignocellulosic substrates, a common method to obtain a higher biodegradability [17]. Thus, also in a view of a possible upscale of the process, the use of CW as co-substrate proved to be a suitable alternative to the physico-chemical pretreatments conventionally used to enhance the biodegradability of the HBRs, thereby avoiding the cost related to chemicals while obtaining VFA yields comparable or higher than those achieved after conventional pretreatments.

### 3.3 Batch fermentation tests for SCP production

**Fig. 5:**
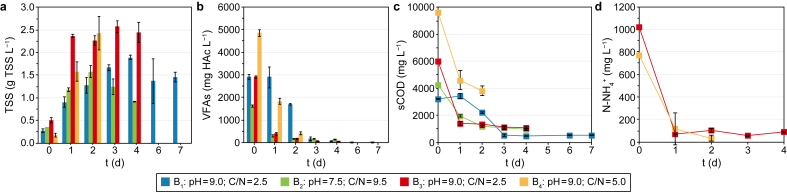
Evolution of the biomass growth in terms of total suspended solids (TSS) (**a**) and consumption of volatile fatty acids (VFAs) (**b**), sCOD (**c**) and ammonium nitrogen (N–NH_4_^+^) (**d**) during the four aerobic fermentation tests aimed at single cell protein (SCP) production. The N–NH_4_^+^ consumption was referred only to B_3_ and B_4_ where the C/N ratio was adjusted by adding N–NH_4_^+^ as nitrogen source. Bars represent the standard deviation of three experimental replicates.

In Fig. 5, the biomass growth in terms of TSS concentration (Fig. 5a) as well as the VFA (Fig. 5b), sCOD (Fig. 5c) and ammonium nitrogen (Fig. 5d) consumption during the four batch aerobic fermentation tests are reported.

The aim of the aerobic tests was not only to investigate the ability of the anaerobic biomass in the liquid VFA-rich fermentate to grow under aerobic conditions but also to evaluate suitable conditions (i.e., C/N ratio, pH, and nitrogen source) to promote SCP production from the VFAs previously produced. During B_1_, the aerobic fermentation led to an almost complete VFA consumption (i.e., 98%) after four days (Fig. 5b) and the highest biomass concentration (i.e., 1.90 g TSS L^−1^) (Fig. 5a), which gradually decreased in the following three days. The VFA concentration trend follows that of sCOD (Fig. 5a), which reached the minimum value (i.e., 461 mg L^−1^) on day four and remained constant, with a sCOD removal of 86%. Concerning the protein content, the percentage of the dry biomass (i.e., g protein per g TSS) was 39% on day four.

Results of B_1_ revealed that the same biomass involved in the anaerobic co-AF process (e.g., LAB), from which the VFA-rich fermentate originated, could also grow and produce SCP under aerobic conditions, thereby valorising the same anaerobic biomass and avoiding the use of other aerobic microorganisms. Moreover, biomass growth in an alkaline environment (i.e., pH = 9.0) is an aspect that should not be underestimated. This condition can prevent SCP contamination by pathogenic microorganisms, which typically proliferate under low pH conditions [66], and limit the competition for substrates with other microbial species, typically growing better in neutral or acidic environments [67].

Given the above, it was decided to reduce the period of the aerobic process to four days in B_2_ and to correct the pH of the liquid fermentate to 7.5 to compare the growth of the same microorganisms under circumneutral conditions, which were reported as more suitable for the CW microorganisms [68]. Furthermore, more neutral pH values could reduce nitrogen losses through ammonia stripping at alkaline pH [69]. The process resulted in a faster biomass growth, which reached its maximum value (i.e., 1.58 g TSS L^−1^) after two days (Fig. 5a), being only 17% lower than the maximum value of B_1_ after four days (i.e., 1.90 g TSS L^−1^), but 19% higher than the B_1_ biomass concentration after two days (i.e., 1.28 g TSS L^−1^). Similarly, VFA consumption reached 89% after only two days (Fig. 5b), coherently with the sCOD removal reaching 72% in the same time interval (Fig. 5c). However, compared to B_1_, B_2_ showed lower biomass yield and protein content performances. In fact, on day two, a protein content of 28% was obtained, which was 28% lower than that of B_1_ on day four (i.e., 39%). Furthermore, the biomass yield on day two (i.e., 0.40 g TSS per g sCOD) was 32% lower than the yield obtained in B_1_ on day four (i.e., 0.59 g TSS per g sCOD). Probably, the higher C/N ratio of B_2_ (i.e., 9.5) set nitrogen limiting conditions, resulting in a lower protein content than B_1_ (i.e., C/N = 2.5). The effect of pH, therefore, was not as relevant to the process as that of C/N.

The influence of different C/N ratios was further investigated in B_3_ and B_4_. B_3_ was carried out under the same operating conditions as B1, but the nitrogen source was changed. Thus, nitrogen in the form of N–NH_4_^+^ was considered in the C/N ratio differently from the Kjeldhal nitrogen (i.e., organic N + N–NH_4_^+^) considered for B_1_ (Table 3). N–NH_4_^+^ was used as a more readily assimilable nitrogen source for the microorganisms involved in the process [68]. The higher initial amount of N–NH_4_^+^ (i.e., 1018 mg L^−1^) (Fig. 5d) seemed to be beneficial for the process in terms of biomass growth. The highest biomass concentration was obtained after three days (i.e., 2.57 g TSS L^−1^) (Fig. 5a), being 26% higher than the maximum value obtained in B_1_ after four days (i.e., 1.90 g TSS L^−1^). Also, the maximum VFA (Fig. 5b) and sCOD consumption (Fig. 5c) were obtained after three days (i.e., 97% and 81%, respectively). Considering N–NH_4_^+^ (Figs. 5d), 962 mg L^−1^ of N–NH_4_^+^ were consumed after one day (i.e., −94%), but only a part was assimilated by the microorganisms as protein. The protein content in the biomass on day three was 33%, corresponding to 137 mg L^−1^ of N-protein (obtained by dividing for the conversion factor 6.25). Thus, only 14% of the N–NH_4_^+^ was eventually converted into protein, while the other 86% was probably lost as stripped nitrogen due to the alkaline pH. Moreover, with a low C/N ratio such as 2.5, the amount of N could exceed the growth and metabolism requirements of the microorganisms, leading to a lower N assimilation [70].

Based on these results, it was decided to increase the C/N ratio (i.e., 5.0) (Table 3) in B_4_ and decrease the duration of the experiment to avoid an excessive stripping of the nitrogen fed. In fact, a typical aerobic fermentation process has a short operating period (e.g., 2–48 h) [5,33]. The test lasted two days, during which the VFAs were almost completely consumed (i.e., 92%) (Fig. 5b), and the biomass showed the highest concentration of 2.43 g TSS L^−1^, which was only 5% lower than the highest biomass concentration of 2.57 g TSS L^−1^ obtained in B_3_ after three days. The sCOD (Fig. 5c) trend was in line with that of VFAs, and a sCOD consumption of 60% was obtained. Despite a lower sCOD consumption if compared to the previous tests, the amount of sCOD removed (i.e., 5801 mg L^−1^) in two days was the highest among all the performed tests, probably due to the highest initial VFA concentration in the liquid fermentate (i.e., 4829 mg HAc L^−1^) (Table 3). Concerning the biomass yield, a value of 0.39 g TSS per g sCOD was obtained in B_4_ after two days, which was only 8% lower than the value obtained in B_3_ (i.e., 0.42 g TSS per g sCOD) and comparable to the yield of B_2_ after two days (i.e., 0.40 g TSS per g sCOD). Contrary, the yield of B_4_ was 34% lower than that obtained in B_1_ after four days (i.e.*,* 0.59 g TSS per g sCOD). The highest biomass concentration obtained in B_4_ could have been attributed mainly to the higher initial sCOD and VFA concentrations in the fermentate (Table 3 and Fig. 5c and d). However, from the nitrogen point of view, B_4_ resulted in the highest protein accumulation. 734 mg L^−1^ of N–NH_4_^+^ were consumed after two days (i.e., *−*96%) (Fig. 5d) and about 22% of this N was eventually converted into protein (i.e., 164 mg L^−1^ of N-protein). As proof of that, the protein content on day two was 42%, which was 21% higher than the content obtained in B_3_ after three days (i.e., 33%) and 7% and 34% higher than the content of B_1_ (i.e.*,* 39%) after four days and B_2_ (i.e., 28%) after two days, respectively.

The conditions used in B_4_ (i.e.*,* C/N = 5.0 and pH = 9.0) seemed to be the best among the tested ones in terms of biomass growth (i.e., 2.43 g TSS L^−1^ in two days) and protein content (i.e., 42% of the dry biomass). The comparison with the protein content obtained in other studies in literature, in which lignocellulosic materials were used, shows that the optimal value obtained in this study (i.e., 42%) was higher or comparable than most of them, which performed the SCP production mostly with pure and dedicated mono- or co-cultures of aerobic filamentous fungi or yeasts. Wang et al. [71] used a mixed solid fermentation of two fungi, such as *Trichoderma reseei* and *Candida tropicalis*, to produce SCP from maise stalk after a physical pretreatment (i.e., steam explosion). The authors obtained a protein content of 32%, 24% lower than in this study. Instead, a 29% lower protein content (i.e., 30%) was obtained by Khan and Dahot [72] from acid (H_2_SO_4_)-pretreated rice husk supplemented with 1.0% sucrose and inoculated with the *Penicillium expansum* fungus. Differently, Rajoka et al. [73] used a yeast such as *Candida utilis* in a batch aerobic fermentation with defatted rice polishings, obtaining a protein content of 38% (i.e., 9% lower than this study). A protein content similar (i.e., 41%) to this study was obtained by Yunus et al. [74] from wheat bran and two yeasts, such as *Candida utilis* and *Rhizopus oligosporus*, after two days of batch aerobic fermentation. On the contrary, a 16% higher protein content (i.e., 50%) was obtained by Banerjee et al. [75] with alkali-pretreated (NaOH) corn stover and *Neurospora sitophila* and a 12% higher protein content (i.e., 48%) was obtained by Nigam [76] from sugar cane bagasse hemicellulosic hydrolysate, after H_2_SO_4_ pretreatment, and *Candida langeronii*. It could be worth noting that all the studies mentioned above used nitrogen sources such as ammonium salts, thus similar to the one added in this study, to grow the different cultures of microorganisms, although working under neutral or acid conditions (i.e., pH = 5.00–7.60), differently from this study. Therefore, having achieved higher average SCP concentrations even working under alkaline pH conditions and with facultative anaerobic mixed microbial cultures is an important aspect that should be emphasised.

## 4 Conclusions

The use of CW allowed to enhance the biodegradability of the HBRs and the VFA production by increasing organic carbon availability and stimulating microbial activity towards HBRs hydrolysis, thus representing a valuable alternative to conventional chemical pretreatments. Up to 3115 mg HAc L^−1^ of VFAs were obtained with an HBR:CW ratio of 4 g VS:g VS. The increase in the HBRs amount led to a proportional increase in the VFA concentration and productivity (i.e., up to 819 mg HAc L^−1^ d^−1^) and in the HBRs hydrolysis because of a decreasing VS/TS ratio. The synergistic effect between the HBRs and CW was further demonstrated by considering that the experimental VFA production was almost 40% higher than that theoretically obtainable from the HBRs and the CW alone.

The VFA-rich fermentate produced in the semi-continuous co-AF process of HBRs and CW proved to be a suitable carbon- and nutrient-containing substrate to sustain the growth of the anaerobic biomass under fully aerobic conditions, producing valuable SCP. With a C/N ratio of 5.0 and a pH of 9.0, up to 92% of the VFA produced, and 60% of the inlet sCOD were converted into 2.43 g TSS L^−1^ with a protein content of 42%, validating the overall feasibility of the VFA-mediated HBRs-to-protein valorisation process.

## CRediT authorship contribution statement

**Carlo Moscariello:** Writing - Review & Editing, Writing - Original Draft, Methodology, Investigation, Data Curation, Conceptualization. **Silvio Matassa:** Writing - Review & Editing, Supervision, Conceptualization. **Francesco Pirozzi:** Writing - Review & Editing, Supervision. **Giovanni Esposito:** Writing - Review & Editing, Supervision. **Stefano Papirio:** Writing - Review & Editing, Supervision, Resources, Project Administration, Funding Acquisition.

## Declaration of competing interest

The authors declare that they have no known competing financial interests or personal relationships that could have appeared to influence the work reported in this paper.
