# Supplementary material for: Valorisation of industrial hemp (Cannabis sativa L.) residues and cheese whey into volatile fatty acids for single cell protein production
Source: Environ Sci Ecotechnol. 2024 Jun 14;21:100439. doi: 10.1016/j.ese.2024.100439 (PMC11254950; doi:10.1016/j.ese.2024.100439)
Supplement: Multimedia component 1 [file mmc1.zip › SUPPLE_spl_1.DOC]

**Valorisation of Industrial Hemp (*Cannabis sativa* L.) Residues and Cheese Whey into Volatile Fatty Acids for Single Cell Protein Production**

Carlo Moscariello^a, *^, Silvio Matassa^a^, Francesco Pirozzi^a^, Giovanni Esposito^a^, Stefano Papirio^a^

^a^ Department of Civil, Architectural and Environmental Engineering, University of Napoli Federico II, via Claudio 21, 80125 Napoli (Italy)

^*^  Corresponding author: Carlo Moscariello, [carlo.moscariello2@unina.it](mailto:carlo.moscariello2@unina.it)

**SUPPLEMENTARY MATERIALS**

**Figure 1S** – Mean biomass concentration in terms of total suspended solids per litre (g TSS L^-1^) along the 137 days of semi-continuous AF of HBRs (i.e., HH and Mix) and CW. Each value represents the mean of the TSS for the corresponding period. Bars represent the standard deviation calculated for each period.

Period I = co-AF of HBRs and CW_A_ with HBRs:CW = 1:1 (g VS g VS^-1^); Period II = AF of HBRs without CW_B_; Period III = co-AF of HBRs and CW_B_ with HBRs:CW = 1:1 (g VS g VS^-1^); Period IV = co-AF of HBRs and CW_B_ with HBRs:CW = 2:1 (g VS g VS^-1^); Period V = co-AF of HBRs and CW_B_ and CW_C_ with HBRs:CW = 4:1 (g VS g VS^-1^).


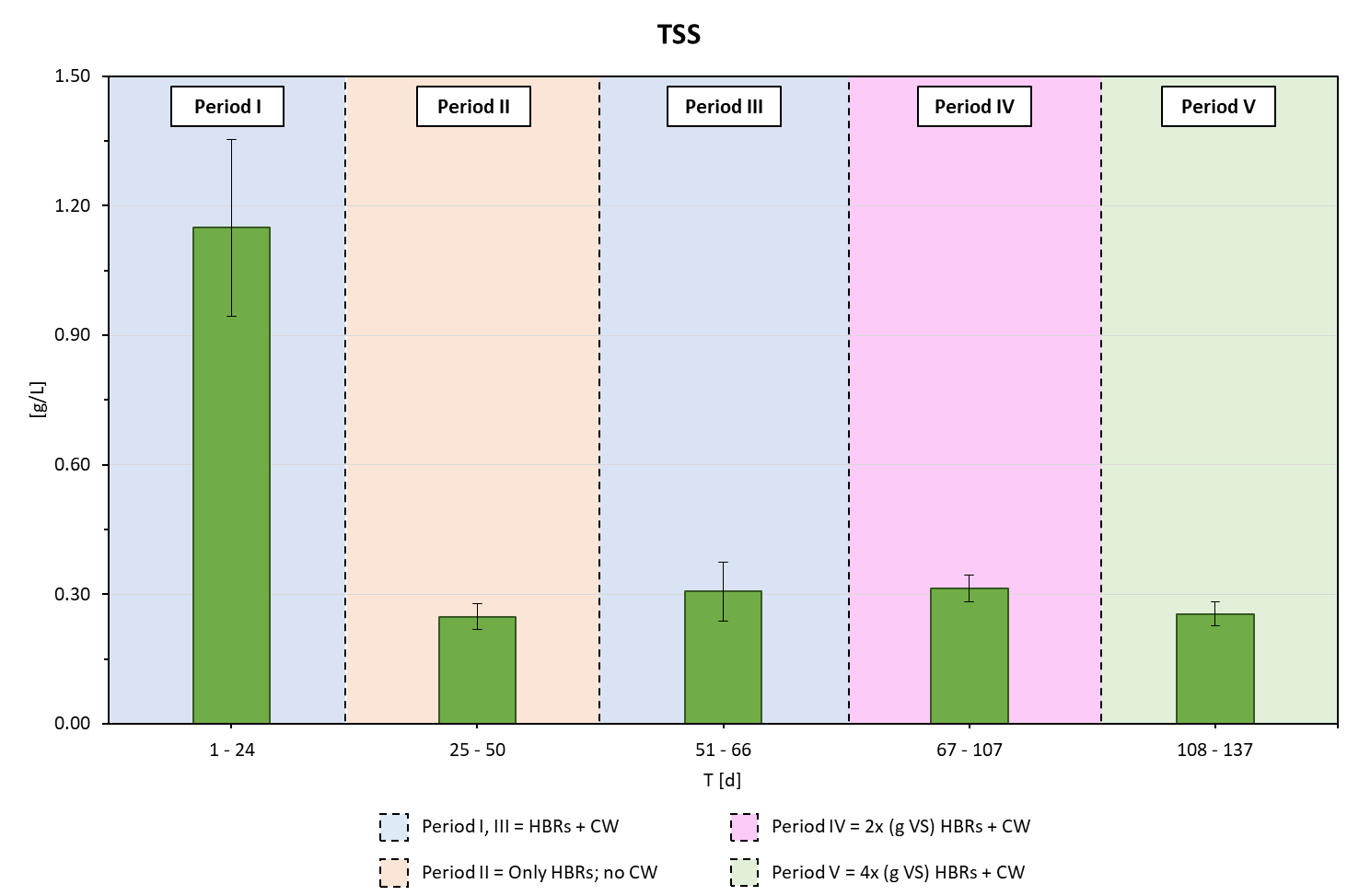

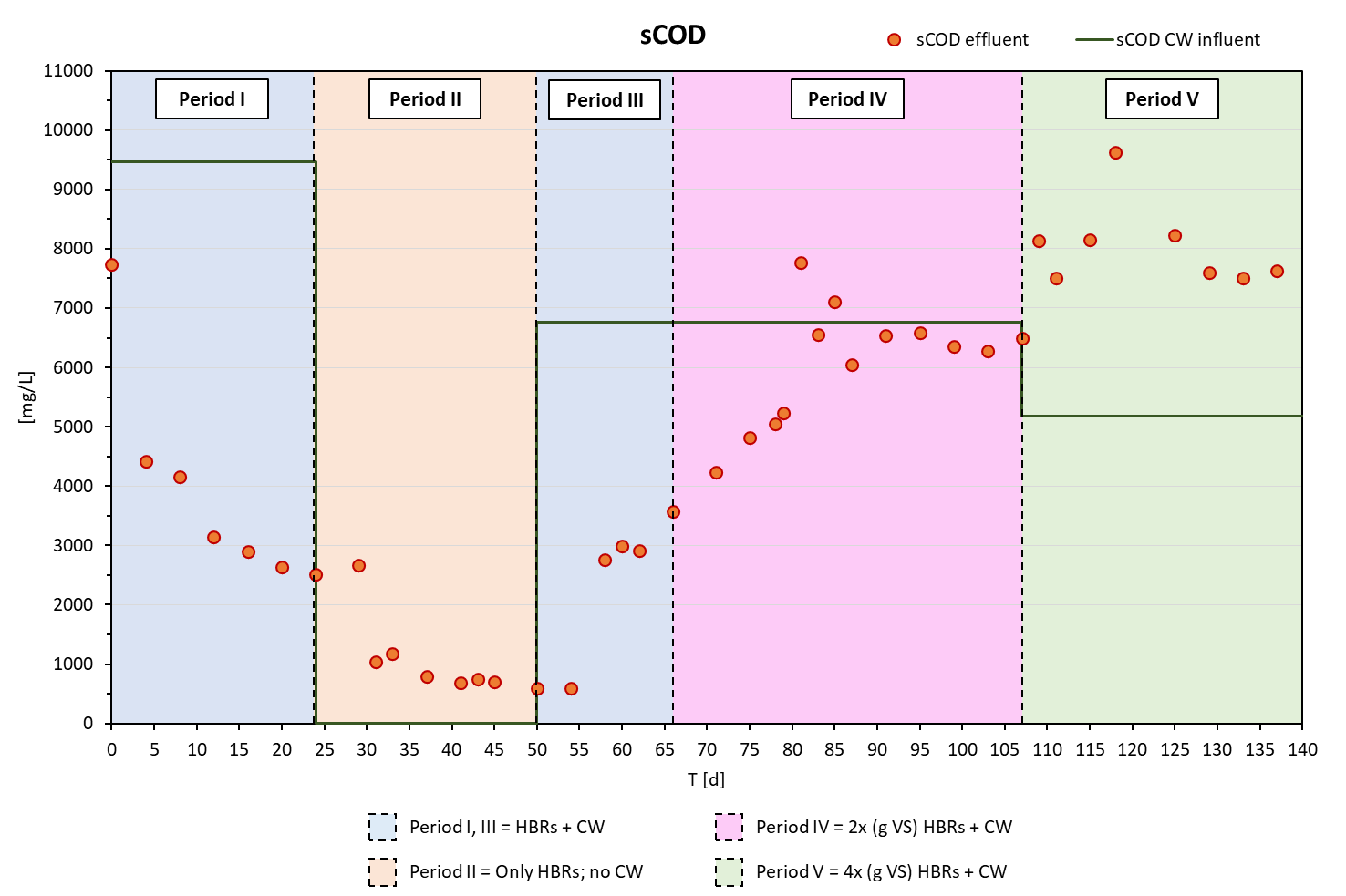


**Figure 2S** - Soluble COD concentration along the 137 days of semi-continuous AF of HBRs (i.e., HH and Mix) and CW. Also, the sCOD of the influent is reported with the green solid line.

Period I = co-AF of HBRs and CW_A_ with HBRs:CW = 1:1 (g VS g VS^-1^); Period II = AF of HBRs without CW_B_; Period III = co-AF of HBRs and CW_B_ with HBRs:CW = 1:1 (g VS g VS^-1^); Period IV = co-AF of HBRs and CW_B_ with HBRs:CW = 2:1 (g VS g VS^-1^); Period V = co-AF of HBRs and CW_B_ and CW_C_ with HBRs:CW = 4:1 (g VS g VS^-1^).

**Table 1S** – Mean sCOD concentration, experimental and theoretical VFA production, and mean VS to TS ratio along the five periods of semi-continuous co-AF between HBRs and CW. Influent COD refers to the sCOD of the CW diluted in tap water. The experimental VFA production refers to the one reported in Figure 3. The theoretical VFA production was calculated as reported in section 2.6. Δ is the difference between the experimental and the theoretical VFA production. TS and VS were measured at the end of each HRT.

| **Periods** | **Days** | **Effluent COD ^a^** | **Influent COD** | **VFA_exp._ ^c^** | **VFA_Theor._** | **Δ** | **VS/TS** |
| --- | --- | --- | --- | --- | --- | --- | --- |
|  |  | **[mg L^-1^]** | **[mg L^-1^]** | **[mg HAc L^-1^]** | **[mg HAc L^-1^]** | **[%]** | **[%]** |
| I | 1 – 24 | 3930 | 9460 | 1202 | 1267 | - 5% | 86.5 |
| II | 25 – 50 | 1050 | n.a. ^*^ | 332 | n.a. | n.a. | 88.5 |
| III | 51 – 66 | 2568 | 6766 | 1332 | 1267 | + 5 % | 84.8 |
| IV | 67 – 107 | 6082 |  | 2235 | 1599 | + 40% | 82.8 |
| V | 108 – 137 | 8046 | 5174 ^b^ | 3115 | 2264 | + 38% | 83.1 |

a = calculated as the mean of the sCOD concentrations measured along each period; b = calculated as mean between the sCOD of the influent with CW_B_ and the one with CW_C_; c = calculated as mean between the VFA production measured along each period (Fig. 3); ^*^n.a. = not available.
